# Prediction of ADHD symptoms from prenatal data in two large population-based cohorts

**DOI:** 10.1192/j.eurpsy.2022.384

**Published:** 2022-09-01

**Authors:** N. Dooley, M. Cannon, D. Cotter, M. Clarke

**Affiliations:** Royal College of Surgeons in Ireland, Psychiatry, Dublin, Ireland

**Keywords:** adhd, Foetal Growth, Prenatal Risks, machine learning

## Abstract

**Introduction:**

The association between low birth weight and attention problems in childhood has been replicated many times (e.g. Momany, Kamradt, & Nikolas, 2018). However birth weight is unlikely the aetiological start-point of this association, as birth weight is itself the product of many prenatal factors e.g. gestational complications, maternal toxin exposure during pregnancy and basic demographics.

**Objectives:**

We explore (1) which prenatal factors best predict attention problems in two independant population-based cohorts of children (2) which associations, if any, are moderated by sex and (3) we report accuracy statistics of our prenatal prediction algorithm for attention problems.

**Methods:**

Participants were children aged 9 from ABCD study from the United States (N > 9,000) and the Growing Up in Ireland (GUI) study from Ireland (N > 6,000). Selected variables included familial pscyhiatric history, maternal smoking during gestation, prescription and non-prescription drug-use during gestation and a variety of gestational complications. All interactions with sex were also included. We used 5-fold cross-validation and elastic net regression (glmnet) to identify the optimal predictors of attention problems (measured by CBCL and SDQ).

**Results:**

Strongest predictors of attention problems in the U.S. cohort included male sex, number of drugs used during pregnancy, number of family members with a history of mental illness, and number of gestational complications. Sex interacted with several of these risks. Protective factors included being a twin/triplet, being Asian, having higher household income and higher parental education level.

**Conclusions:**

Several risk factors for childhood attention problems were identified across both cohorts, supporting their generalizabilty. Other findings were cohort-specific.

**Disclosure:**

No significant relationships.

